# Development and validation of a novel diagnostic tool for predicting the malignancy probability of thyroid nodules: A retrospective study based on clinical, B-mode, color doppler and elastographic ultrasonographic characteristics

**DOI:** 10.3389/fendo.2022.966572

**Published:** 2022-09-20

**Authors:** Shangyan Xu, Xiaofeng Ni, Wei Zhou, Weiwei Zhan, Huan Zhang

**Affiliations:** ^1^ Department of Ultrasound, Ruijin Hospital, Shanghai Jiao Tong University School of Medicine, Shanghai, China; ^2^ Department of Radiology, Ruijin Hospital, Shanghai Jiao Tong University School of Medicine, Shanghai, China

**Keywords:** thyroid nodule, ultrasonography, clinical, risk factor, nomogram

## Abstract

**Background:**

Clinicians estimate the risk of thyroid nodules and make subsequently decision on the basis of clinical and ultrasonographic findings. Currently, there is no comprehensive diagnostic tool for predicting the malignancy rates of thyroid nodules. Our aim was to develop and validate a novel integrate diagnostic tool for predicting the malignancy probability of thyroid nodules based on clinical, B-mode, Color Doppler and elastographic ultrasonographic characteristics.

**Methods:**

A total of 1016 nodules in 1016 patients who underwent thyroid ultrasonography and surgery from July 2021 to December 2021 were included in this retrospective study. All nodules were confirmed by pathology and randomly classified into the training and validation groups. Clinical, B-mode, Color Doppler and elastographic (CBCE) ultrasonographic characteristics of nodules were recorded. Univariate and multivariate analyses were performed to screen independent predictors associated with thyroid cancer. A multivariate model containing the extracted predictors was constructed and presented in the form of a nomogram. The validation and applicability of the CBCE nomogram was evaluated using the receiver operating characteristic (ROC) curve. Diagnostic performances were calculated to compare the CBCE nomogram with ACR-TIRADS (Thyroid Imaging Reporting Data System by American College of Radiology) and EU-TIRADS (Thyroid Imaging Reporting Data System by European Thyroid Association).

**Results:**

The following factors were included in the CBCE nomogram: patient gender, age, shape, margin, composition and echogenicity, calcification, vascularization distribution, vascularization degree, suspicious lymph node metastases and elastography. The area under the curve (AUC) values were 0.978 and 0.983 for the training and validation groups, respectively. Compared with ACR-TIRADS and EU-TIRADS, the CBCE nomogram showed improved accuracy (0.944) and specificity (0.913) without sacrificing sensitivity (0.963) and showed the highest AUC with an optimal cutoff value of 0.55.

**Conclusion:**

The CBCE nomogram has good and high clinical practicability in predicting the malignancy probability of thyroid nodules.

## Introduction

Thyroid nodules (TNs) are very common (1, 2). There are several factors that can affect their prevalence, including demographic characteristics, iodine sufficiency status and the increasing use of ultrasound (US) examination (3–5). In recent years, overdiagnosis and overtreatment of TNs have become a global problem (6, 7). Correct differentiation between low-risk and high-risk TNs is a crucial starting point for optimal treatment (4). Ultrasonography, as a radiation-free and non-invasive method, is the first-line approach for thyroid examination. The US-based risk stratification systems (RSSs) of TNs, such as ACR-TIRADS (Thyroid Imaging Reporting Data System by American College of Radiology) (8) and EU-TIRADS (Thyroid Imaging Reporting Data System by European Thyroid Association) (9), have remained a research focus for nearly a decade. Numerous studies have verified and compared their diagnostic performances, showing good values in clinical practice but also problems of complex use and weak consistency (10–15).

Moreover, clinicians estimate the risk of TNs and make subsequent decisions based on comprehensive information including clinical and US findings (3, 16, 17). The existing RSSs are limited to B-mode US features and do not address clinical, Color Doppler and elastographic US characteristics. Previous studies have shown that there were gender and age differences in patients with thyroid cancer (TC), but this is still controversial in recent years (16, 18). In addition, routine TSH (Thyroid Stimulating Hormone) measurement is recommended by the ATA (American Thyroid Association) guideline which not only aimed to exclude hyperthyroidism but to better stratify the risk of malignancy as well, since higher serum TSH levels have been correlated with an increased risk of malignancy (19). Furthermore, vascularity information and elastography techniques have been seen as complementary imaging modalities for the diagnosis of TNs (20). However, there is no integrated diagnostic tool for TNs currently.

This research aims to develop and validate a comprehensive and easy-to-use diagnostic tool for TNs based on clinical, B-mode, Color Doppler and elastographic US characteristics, providing more information for clinicians as well as avoiding overdiagnosis and overtreatment.

## Materials and methods

### Patients selection

**Figure 1:**
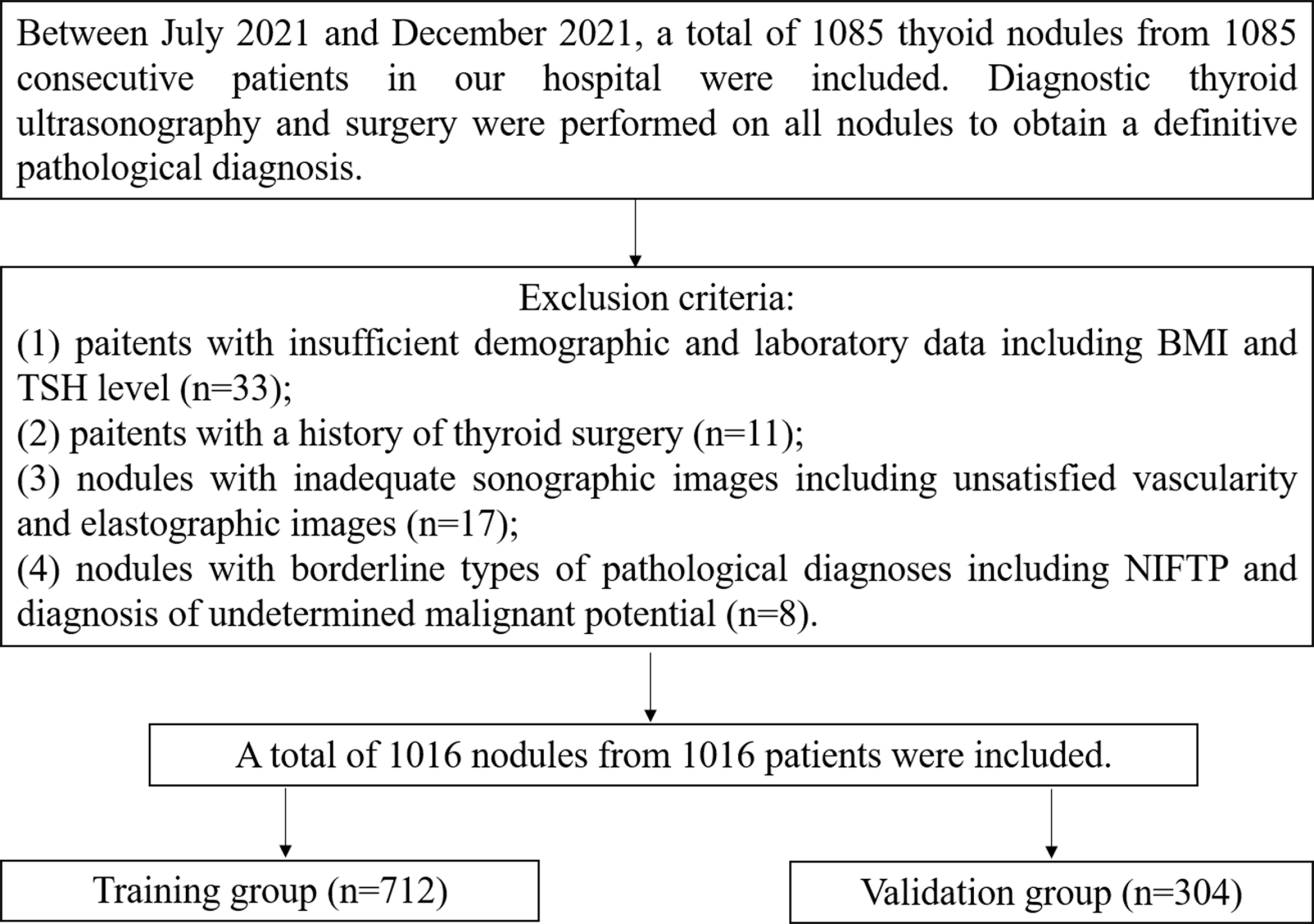
The study flow chart.

This retrospective study was approved by the Ethics Committee of Ruijin Hospital, Shanghai Jiao Tong University School of Medicine, and the requirement for written informed consent was waived. Between July 2021 and December 2021, a total of 1085 thyoid nodules from 1085 consecutive patients in our hospital were included. Diagnostic thyroid ultrasonography and surgery were performed on all nodules to obtain a definitive pathological diagnosis. Among them, 69 nodules were excluded. The exclusion criteria were as follows (1): patients with insufficient demographic and laboratory data including BMI (Body Mass Index) and preoperative TSH level (n=33) (2); patients with a history of thyroid surgery (n=11) (3); nodules with inadequate sonographic images including unsatisfied vascularity and elastographic images (n=17) (4); nodules with borderline types of pathological diagnoses including NIFTP (Noninvasive follicular thyroid neoplasm with papillary-like nuclear features) and diagnosis of undetermined malignant potential. (n=8). Ultimately, a total of 1016 nodules from 1016 patients were included. All nodules were randomly divided into the training group (n=712) and the validation group (n=304) at a ratio of 7:3. The training group was used to build the nomogram, while the validation group was used to evaluate the diagnostic performance of this tool. The study flow chart is shown in **Figure 1**.

### Clinical data acquisition

Clinical data were gathered by searching the medical records of patients gender, age, BMI, residence, family history of TC and preoperative TSH level. Residences were divided into inland areas and coastal areas. Family history of TC was defined as a first-degree relative with a history of TC. Preoperative TSH level was defined as the test results within one week before surgery. The normal range of TSH levels in our institution is 0.35–4.94 µIU/ml.

### Ultrasound image acquisition

Two radiologists (S.Y.X and X.F.N) with over ten years of experience in thyroid sonography used a 4-15 MHz linear probe (MyLab9, Esaote, Italy) to perform all grayscale, color Doppler and elastography sonographic examinations. Images in the longitudinal and transverse directions of each target nodule were obtained. A picture archive and communication system recorded and uploaded all the images for later retrospective analysis. The grayscale, color Doppler and elastography sonographic features of the target nodules were assessed by two radiologists (S.Y.X and X.F.N) with professional training in thyroid ultrasound in consensus. In the case of disagreement between the two radiologists, the final decision was made by a third radiologist (W.Z) with 20 years of experience in thyroid ultrasound.

The B-mode, color Doppler US features of TNs included shape (wider-than-tall, taller-than-wide), margin (regular, irregular), composition and echogenicity (non-solid hypoechoic, solid hypoechoic), calcification (non-microcalcification, microcalcification), vascularization distribution (internal, non-internal), vascularization (low, high), diffuse lesion (absent, present) and suspicious lymph node metastases (LNM) (absent, present) in the cervical compartment. The vascularization distribution is divided into types 1-4 (type 4, marked intranodular vascularity with or without perinodular vascularity (vascularity≥50%); type 3: mild intranodular vascularity with or without perinodular vascularity (vascularity<50%); type 2: perinodular vascularity only; type 1: no vascularity) (20). In our study, types 3 and 4 were classified as the group with internal vascularity, while types 1 and 2 were classified as the group with non-internal vascularity. The vascularity greater than or similar to that of the surrounding thyroid tissue indicated a high vascularization degree of a nodule (21). The rest of the nodules were relatively low. Diffuse lesions referred to a thyroid US appearance consistent with a diffuse disease (e.g. chronic thyroiditis or lymphoma). Ultrasonographic features of metastatic lymph nodes included the absence of a hilum, a Solbiati index <2, peripheral vascular flow, hyperechoic, calcifications, and cystic (22). Based on the 2013 European Thyroid Association Guidelines for cervical ultrasound scan (23), cervical LNs can be classified into three groups: normal, indeterminate and suspicious for malignancy. In our study, we classified the latter two as the group with suspicious LNM and the rest as the group with no suspicious LNM. Elastographic US features were classified as soft and hard by the strain elastography technique according to the Asteria criteria (24). Asteria criteria have four scores of tissue stiffness: 1 and 2 for soft nodules and 3 and 4 for hard lesions.

### Statistical analysis

Continuous variables are expressed as the mean ± SD. The variances were equal or unequal for continuous data using Student’s t test. The Mann–Whitney U test was used for continuous data with a non-normal distribution. Categorical variables were expressed as numbers (%), and categorical data were evaluated by Fisher’s exact test and Pearson’s χ2. Univariate and multivariate analyses were performed to screen independent predictors associated with TC. A multivariate logistic model containing the extracted predictors was constructed and presented in the form of a nomogram.

To measure the accuracy of the nomogram, the Hosmer–Lemeshow goodness-of-fit test was applied. To validate the nomogram, two ROC curves were produced for the training and validation groups. Diagnostic values were calculated to assess the applicability of the nomogram and to compare it with ACR-TIRADS (8) and EU-TIRADS (9). Both these two RSSs are divided into five grades according to different US characteristics, which classified as TR1-5 for ACR-TIRADS and EU-TIRADS 1-5 for EU-TIRADS. The comparasion of diagnostic performances for the CBCE nomogram, ACR-TIRADS and EU-TIRADS were based on the AUC, sensitivity, specificity, positive predictive value (PPV), negative predictive value (NPV), and accuracy. The analysis was performed using the SPSS software (version 23; IBM Corp., Armonk, NY, USA) and R software (version 4.1.0; R Development Core Team, Vienna, Austria). A *P* value < 0.05 was considered for significant differences.

## Results

### Baseline for patients with thyroid nodules

Among the 1016 thyroid nodules, 374 were benign and 642 were malignant. The average maximum diameters were 34.15 ± 17.16 mm for benign nodules and 10.23 ± 7.38 mm for malignant nodules. Follicular adenoma (n=22), Hashimoto thyroiditis (n=39), and nodular goiter (n=313) were benign lesions. Malignant lesions included follicular thyroid carcinoma (n=10), medullary thyroid carcinoma (n=15), and papillary thyroid carcinoma (n=617).

### Univariate and multivariate analyses of risk factors for patients with thyroid nodules

The univariate and multivariate analyses results were showed in **Table 1** and **Table 2**. Except for the three indicators of BMI, family history of TC and TSH, the remaining indicators showed significant differences in the training group (*P* < 0.05). **Table 2** showed the extracted independent predictors included gender, age, shape, margin, composition and echogenicity, calcification, vascularization distribution, vascularization degree, and suspicious LNM and elastography based on the training group (*P* < 0.05). These ten features were included into the development of the nomogram associated with TC.

**Table 1:** Univariate analysis of risk factors for thyroid cancer in the training group.

Factors	Training group (n=712)		
	Benign (n=259)	Malignant (n=453)	*P* value
**Gender (%)**			<0.001
Female	225 (86.9)	282 (62.3)	
Male	34 (13.1)	171 (37.7)	
**Age (mean ± SD)**	49.25 ± 13.06	41.66 ± 10.73	<0.001
**Residence (%)**			<0.001
inland area	66 (25.5)	204 (45.0)	
coastal area	193 (74.5)	249 (55.0)	
**BMI (mean ± SD)**	23.20 ± 3.08	23.52 ± 3.62	0.216
**Family History of Thyroid Cancer (%)**			0.141
No	238 (91.9)	429 (94.7)	
Yes	21 (8.1)	24 (5.3)	
**TSH (mean ± SD), µIU/mL**	1.55 ± 1.40	1.85 ± 1.25	0.103
**Shape (%)**			<0.001
wider-than-tall	242 (93.4)	181 (40.0)	
taller-than-wide	17 (6.6)	272 (60.0)	
**Margin (%)**			<0.001
regular	234 (90.3)	24 (5.3)	
irregular	25 (9.7)	429 (94.7)	
**Composition and Echogenicity (%)**			<0.001
non-solid hypoechoic	189 (73.0)	16 (3.5)	
solid hypoechoic	70 (27.0)	437 (96.5)	
**Calcification (%)**			<0.001
non-microcalcification	227 (87.6)	157 (34.7)	
microcalcification	32 (12.4)	296 (65.3)	
**Vascularization Distribution (%)**			0.007
non-internal	149 (57.5)	213 (47.0)	
internal	110 (42.5)	240 (53.0)	
**Vascularization Degree (%)**			<0.001
low	136 (52.5)	373 (82.3)	
high	123 (47.5)	80 (17.7)	
**With Diffuse Lesion (%)**			<0.001
No	233 (90.0)	356 (78.6)	
Yes	26 (10.0)	97 (21.4)	
**Suspicious LNM (%)**			<0.001
No	256 (98.8)	312 (68.9)	
Yes	3 (1.2)	141 (31.1)	
**Elastography (%)**			<0.001
soft	188 (72.6)	45 (9.9)	
hard	71 (17.4)	408 (91.1)	

SD, standard deviation; TSH, Thyroid Stimulating Hormone; LNM, lymph node metastases.

**Table 2:** Multivariate Analysis of risk factors for thyroid cancer in the training group.

Risk Factors	Value assignment	B	Standard Error	*P* Value	OR	OR (95% CI)
Gender	0=Female, 1=Male	1.126	0.449	0.012	3.084	1.280, 7.433
Age	continuous variables	-0.028	0.015	0.049	0.973	0.944, 1.002
Shape	0=wider-than-tall, 1=taller-than-wide	1.462	0.450	0.001	4.314	1.785, 10.426
Margin	0=regular, 1=irregular	2.457	0.422	0.000	11.666	5.099, 26.688
Composition and Echogenicity	0=non-solid hypoechoic, 1=solid hypoechoic	1.353	0.517	0.009	3.870	1.405, 10.661
Calcification	0=non-microcalcification, 1=microcalcification	1.406	0.378	0.000	4.079	1.946, 8.551
Vascularization Distribution	0=non-internal, 1=internal	1.267	0.506	0.012	3.551	1.319, 9.566
Vascularization Degree	0=low, 1=high	-1.739	0.529	0.001	0.176	0.062, 0.496
Suspicious LNM	0=No, 1=Yes	2.543	0.843	0.003	12.714	2.436, 66.352
Elastography	0=soft, 1=hard	1.822	0.431	0.000	6.184	2.655, 14.404

LNM, lymph node metastases; OR, Odds Ratio; CI, Confidence Interval.

### Development and validation of the CBCE nomogram

**Figure 2:**
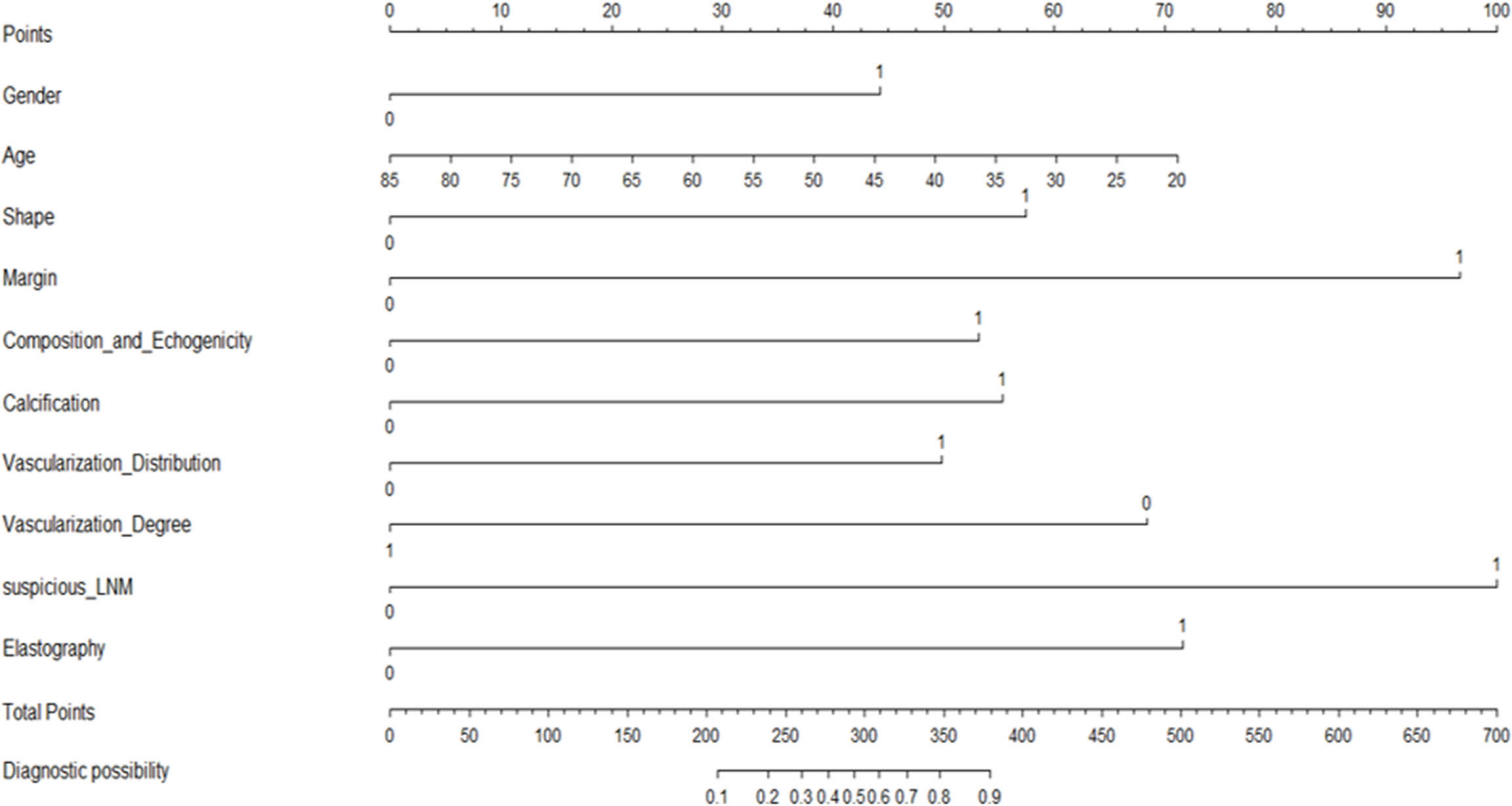
A CBCE nomogram to predict the malignancy probability of thyroid nodules based on clinical, B-mode, Color Doppler and elastographic ultrasonographic characteristics.

**Figure 3:**
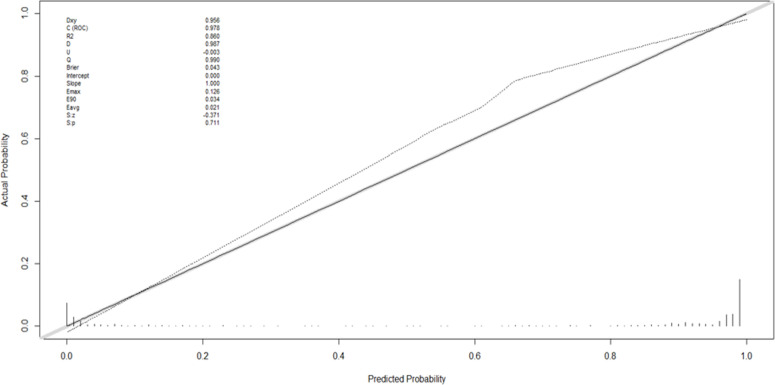
The Hosmer-Lemeshow goodness-of-fit test for the CBCE nomogram.

**Figure 4:**
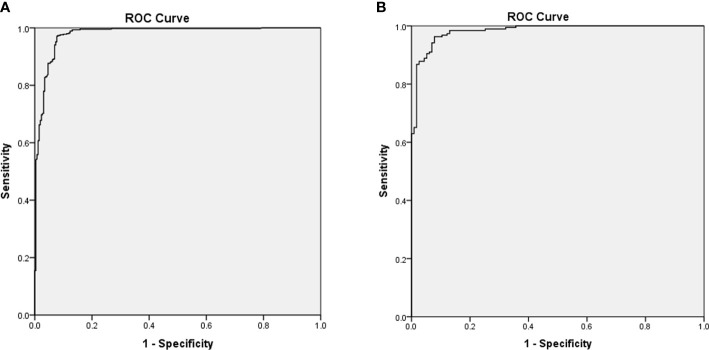
Receiver operating characteristic curves of the training group **(A)** and the validation group **(B)** based on the pathological diagnosis. The area under the curves for the two groups were 0.978 (0.967,0.989) and 0.983 (0.971,0.994), respectively.

**Figure 2** presented the multivariate logistic model as a nomogram with the ten independent predictors. The risk score ranged from a minimum of 0 to a maximum of 700. The corresponding malignancy probability of each nodule was obtained by adding up the specific point of each predictor. From **Figure 3**, the nomogram calibration curve showed floating around the baseline by the Hosmer–Lemeshow goodness-of-fit test. **Figure 4** presented two ROC curves of the training group (A) and the validation group (B). The AUCs for the two groups were 0.978 (95% CI [0.967,0.989]) and 0.983 (95% CI [0.971,0.994]), respectively.

### Diagnostic performances of the CBCE nomogram

**Figure 5:**
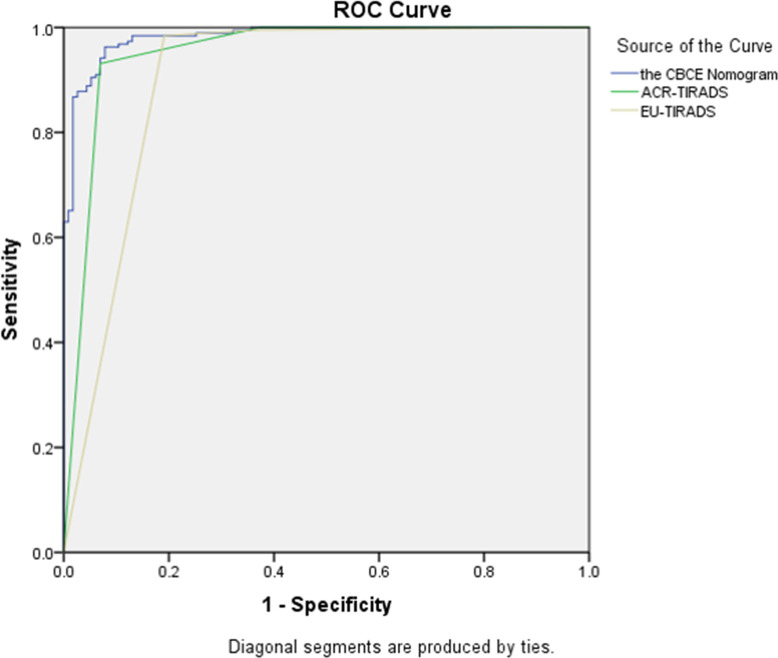
The comparison of Receiver operating characteristic curves among the CBCE nomogram, ACR-TIRADS and EU-TIRADS based on the validation group. The area under the curves for the three models were 0.983 (0.971, 0.994), 0.948 (0.918, 0.978) and 0.889 (0.844, 0.934), respectively.

**Figure 5** showed the CBCE nomogram showed the highest AUC of 0.983 in comparison to ACR-TIRADS (AUC of 0.948) and EU-TIRADS (AUC of 0.889) in the validation group. The optimum cutoff values of the three models were 0.55, TR4 and EU-TIRADS 4, respectively. The numbers of cases according to the cut-off value in the three models are listed in **Table 3**. **Table 4** showed the comparison of diagnostic performances among the CBCE nomogram, ACR-TIRADS and EU-TIRADS based on the validation group, including sensitivity (96.3% vs 98.4% vs 97.4%), specificity (91.3% vs 62.6% vs 64.3%), PPV (94.8% vs 81.2% vs 81.8%), NPV (93.8% vs 96.0% vs 93.7%), and accuracy (94.4% vs 84.9% vs 84.9%). The CBCE nomogram showed the highest specificity, PPV and accuracy, while ACR-TIRADS and EU-TIRADS showed slightly higher sensitivity and NPV.

**Table 3:** Numbers of cases according to the cut-off values in the three models based on the validation group.

Diagnostic Models		Malignant	Benign	Total
the CBCE Nomogram	≥0.55	182	10	192
	<0.55	7	105	112
ACR-TIRADS	TR4-5	186	43	229
	TR1-3	3	72	75
EU-TIRADS	EU-TIRADS 4-5	184	41	225
	EU-TIRADS 1-3	5	74	79
Total		189	115	304

**Table 4:** Diagnostic performances of the CBCE Nomogram, ACR-TIRADS and EU-TIRADS in the validation group.

Diagnostic Models	the cut-off values	AUC (95% CI)	Sensitivity (%)	Specificity (%)	PPV (%)	NPV (%)	Accuracy (%)
the CBCE Nomogram	0.55	0.983 (0.971, 0.994)	0.963 (182/189)	0.913 (105/115)	0.948 (182/192)	0.938 (105/112)	0.944 (287/304)
ACR-TIRADS	TR4	0.948 (0.918, 0.978)	0.984(186/189)	0.626 (72/115)	0.812 (186/229)	0.960 (72/75)	0.849 (258/304)
EU-TIRADS	EU-TIRADS 4	0.889 (0.844, 0.934)	0.974 (184/189)	0.643 (74/115)	0.818 (184/225)	0.937(74/79)	0.849 (258/304)

AUC, area under the curve; PPV, positive predictive value; NPV, negative predictive value.

### Clinical application of the CBCE nomogram

**Figure 6:**
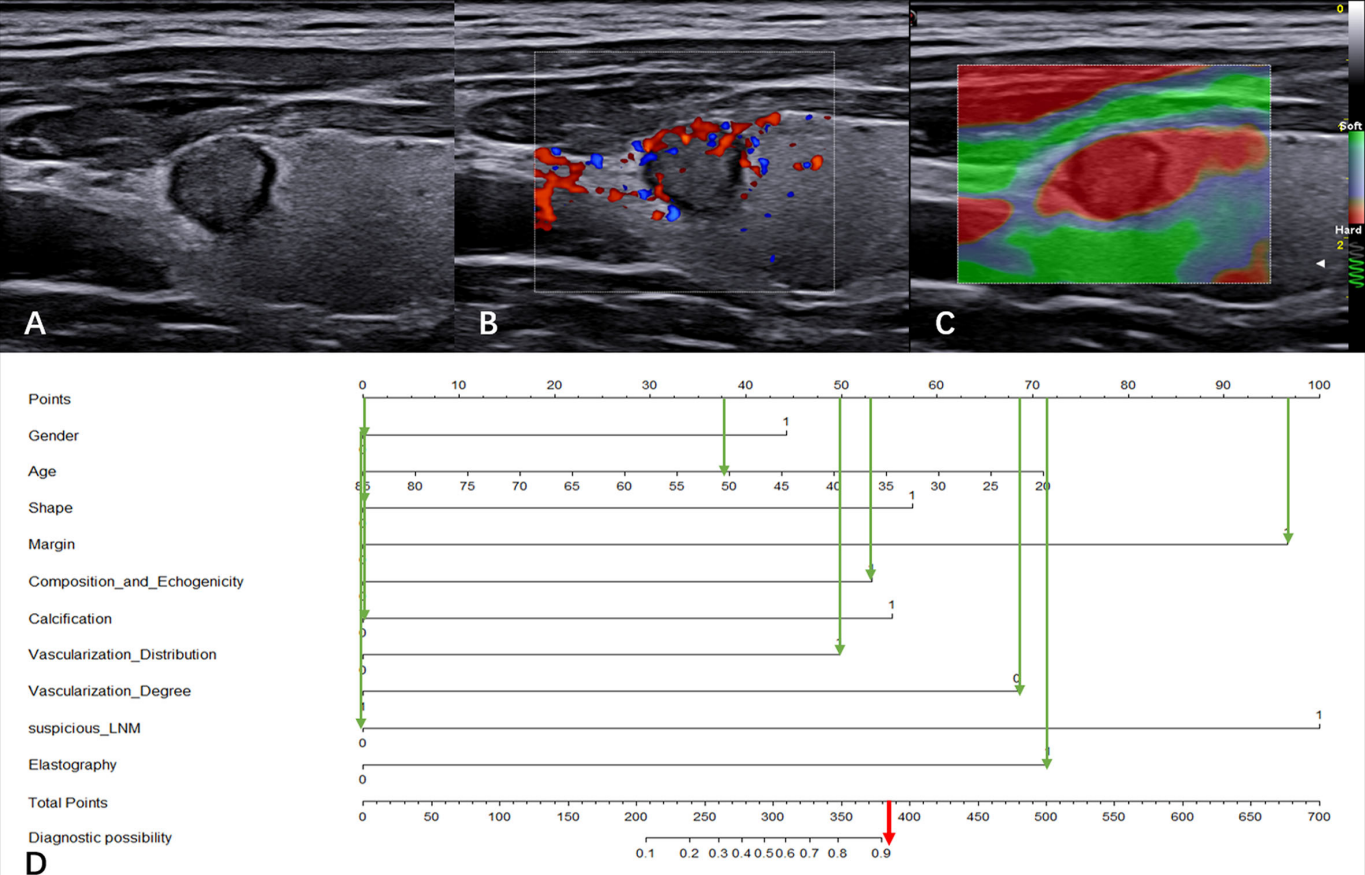
Clinical application of the CBCE nomogram. Images from a 51-year-old woman with a nodule (9.1×7.8×8.2 mm) in the left thyroid and the pathology was papillary thyroid carcinoma. **(A)** Greyscale ultrasound imaging of the mass. The lesion showed a wider-than-taller shape, irregular margins and solid hypoechoicity. **(B)** Internal vascularization distribution and low vascularization degree of the nodule in color Doppler. **(C)** Strain elasticity of the nodule was hard. **(D)** In the nomogram, the total score was 379, and the corresponding malignancy rate of the nodule was high (>0.90). The green arrows indicate the score corresponding to each risk factor, and the red arrow indicates the total score of the nodule.

**Figure 6** illustrates a typical clinical application of the CBCE nomogram. Images were obtained from a 51-year-old woman with a nodule in the left thyroid. The nomogram scored 0 for woman, 38 for age, 0 for wider than taller shape, 97 for irregular margin, 53 for solid hypoechoic, 0 for non-microcalcification, 50 for internal vascularization distribution, 69 for low vascularization degree, 0 for no suspicious LNM, and 72 for hard strain elastography, resulting in a total score of 379 points. The corresponding malignancy rate of the nodule was high (>0.90), and the pathology of the nodule was papillary thyroid carcinoma.

## Discussion

In this study, a novel integrated diagnostic tool called the CBCE nomogram was developed and validated for predicting the malignancy probability of TNs based on clinical, B-mode, Color Doppler and elastographic US characteristics. The CBCE nomogram is not only based on B-mode US features, but also includes clinical, vascularity and elastographic US characteristics, which can provide more information on TNs. Compared with ACR-TIRADS and EU-TIRADS, the CBCE nomogram showed improved accuracy (0.944) and specificity (0.913) without sacrificing sensitivity (0.963) for predicting malignancy. With the optimum cutoff value of 0.55, the CBCE nomogram showed the highest AUC of 0.983 in the validation group. This means that when the malignancy probability of a nodule is higher than 0.55, it is regarded as a high-risk nodule and FNA (fine needle aspiration) is recommended; when the malignant probability of a nodule is lower than 0.55, it is regarded as a low-risk nodule and follow-up ultrasonography is recommended. The significance of this study is to provide a new strategy for clinicians as well as to avoid overdiagnosis and overtreatment.

The clinical characteristics plays an important role in the diagnosis and treatment of patients with TNs. Some studies suggested that thyroid cancer has been more common in women than men, which may related to sexual hormones, although this has been controversial (25, 26). In developed countries, the majority of newly diagnosed thyroid carcinomas correspond to incidentally found papillary microcarcinomas (25). Women are traditionally more keen on undergoing routine clinical examination along their lives as compared to men (27, 28), which could be part of the reasons why thyroid nodules and cancers are more frequently detected in the female population. However, another studies showed that male gender was the clinical factor associated with the high risk of TC (16, 29). Our results showed that the number of TC was more in male than in female, and gender was an independent risk factor associated with TC. These different results may depend on differences in inclusion criteria and in patient’s lifestyle. In terms of age, recent evidence suggested that clinically silent thyroid cancer was frequently present in younger age (30), indicating that screening (31, 32) and environmental factors (33) played a key role in the increased incidence. Our results showed that younger age was also a risk factor associated with TC, similar to previous studies. Other clinical risk factors for TC included a family history of TC, obesity and thyroid dysfunctions (19, 34), but the relevant evidence is uncertain. Our results showed that family history, BMI and TSH level were not independent risk factors associated with TC. Discrepancies across different studies may be due to population samples from different countries and cities. Adding clinical characteristics makes our nomogram more comprehensive and more applicable to clinical practice.

Thyroid ultrasonography is the preferred tool for thyroid imaging because it can clarify the location, size, shape, echogenicity, composition, margin, calcification and vascularity of nodules, as well as the features of cervical lymph nodes. Results (9, 35–41) consistent with malignancy contain hypo-echogenicity, solid, irregular margin, taller-than-wide shape, microcalcification and the presence of suspicious lymph nodes. No single characteristic has been proven to reliably distinguish between benign and malignant lesions. The results also showed that the above conventional US indicators are risk factors associated with TC. The value of vascularity for TNs is different in different studies. Internal vascularity was observed in 30.7%-65.3% of benign nodules and 26.7%-91.7% of malignant nodules (42–46). In terms of the vascularity degree, a study showed a high probability (42%) of malignancy in hypervascular nodules (43), while another study indicated that hypovascular vascularity was frequently observed in malignant nodules (47). In our study, internal vascularization distribution and low vascularization degree were more common in malignant nodules, and these two vascularization features were independent risk factors for TC. Different US instruments have different sensitivities for vascularity detection, which may be related to disparate results. US elastography provides conventional US with complementary information in many human organs. The combined use of elastography with conventional US is able to improve the discrimination of malignant and benign nodules (48). Strain elastography is useful to assess malignancy, with an average specificity of 90% and an overall average sensitivity of 92%, according to a meta-analysis of 639 TNs (49). The findings of this study indicated that the strain imaging of elasticity was an independent risk factor associated with TC. It is worth noting that recently, the International Thyroid Nodule Ultrasound Working Group has been trying to develop a uniform international guideline. The organization proposed extending standardized ultrasonography to elastography of the stiffness in TNs (50). Our research established a complementary role of US elastography and vascularity in the risk stratification of TNs.

The US-based RSSs of TNs provides clinicians with an estimated malignancy rate based on the category. Currently, B-mode US-based RSSs include ACR-TIRADS (8), ATA (19), KTA-TIRADS (Thyroid Imaging Reporting Data System by Korean Thyroid Association) (20), and EU-TIRADS (9). However, there are considerable discrepancies among these RSSs regarding the US features used for risk categories, expected malignancy risk, diagnostic performance, and the size threshold for biopsy. A comparative study of 902 nodules showed that both ATA and KTA­TIRADS had higher sensitivity for the diagnosis of TC but lower specificity than ACR-TIRADS (10). Another comparative study of 3,422 nodules showed that ATA and K-TIRADS had limitations that included no category for all available nodules, and approximately 10% of these uncategorized nodules had malignancy (12). Another study containing the four RSSs mentioned above showed that the interobserver agreement remained only fair to moderate (51). The reasons for these differences may be due to the inconsistent classification of US features and the complexity of the system. Therefore, each predictor was classified into two categories to establish an easy-to-use model in our study. The CBCE nomogram we developed had good diagnostic performances and calibration for the probability of TC. Compared with ACR-TIRADS and EU-TIRADS, the CBCE nomogram showed improved accuracy and specificity without sacrificing sensitivity. Furthermore, the CBCE nomogram showed the highest AUC with the optimum cutoff value of 0.55. A nodule is considered high-risk and FNA is recommended when its malignancy probability is over 0.55, while a nodule is considered low-risk and US follow-up is recommended when its malignancy probability is lower than 0.55. The CBCE nomogram provided a novel and simple diagnostic tool for clinicians.

There are some limitations in this study. First, as findings from a single center, our results have limited generalizability. Although our study has been internally validated and showed good diagnostic performance, it needs to further external validation. Second, as this is a retrospective study, data bias is inevitable. Further prospective studies will be needed to improve the model. Third, our study does not address FNA thresholds for thyroid nodules, which requires further research in the future. Despite these limitations, **our study has several highlights.** First, to our knowledge, this is the first study to develop and validate an integrated diagnostic tool for predicting the malignancy probability of TNs based on clinical, B-mode, Color Doppler and elastographic US characteristics. Second, the CBCE nomogram has been demonstrated to have better diagnostic performances in predicting malignancy than ACR-TIRADS and EU-TIRADS. Third, the CBCE nomogram is an easy-to-use diagnostic tool for predicting malignancy. The corresponding malignancy probability of each nodule will be obtained by adding up the specific points of each predictor. Fourth, our study obtained an optimal cut-off value of 0.55 for distinguishing low-risk from high-risk nodules. The cut-off value separates the thyroid nodules into two groups, one of which is high risk with a malignancy probability above 0.55 and the other of which is low risk with a malignancy probability below 0.55. FNA and US follow-up are recommended for the two groups, separately. The significance of this study is to provide a new strategy for clinicians.

## Conclusions

In conclusion, this study developed and validated a novel, comprehensive and reliable diagnostic tool based on clinical, B-mode, Color Doppler and elastographic ultrasonographic characteristics for thyroid nodules. Our research has two potential benefits, one of which is to identify patients with high-risk nodules who are the candidates for FNA, and the other is to provide a new strategy for clinical practice.

## Data availability statement

The raw data supporting the conclusions of this article will be made available by the authors, without undue reservation.

## Ethics statement

The studies involving human participants were reviewed and approved by the Ethics Committee of Ruijin Hospital, Shanghai Jiao Tong University School of Medicine. Written informed consent for participation was not required for this study in accordance with the national legislation and the institutional requirements.

## Author contributions

All authors contributed to the study conception and design. Material preparation, data collection and analysis were performed by SX and XN. The first draft of the manuscript was written by SX and all authors commented on previous versions of the manuscript. All authors contributed to the article and approved the submitted version.

## Acknowledgments

The authors are grateful to all participants of this study.

## Conflict of interest

The authors declare that the research was conducted in the absence of any commercial or financial relationships that could be construed as a potential conflict of interest.

## Publisher’s note

All claims expressed in this article are solely those of the authors and do not necessarily represent those of their affiliated organizations, or those of the publisher, the editors and the reviewers. Any product that may be evaluated in this article, or claim that may be made by its manufacturer, is not guaranteed or endorsed by the publisher.

## Abbreviations

AUC, Area Under the Curve; ATA, American Thyroid Association; ACR-TIRADS, Thyroid Imaging Reporting Data System by American College of Radiology; BMI, Body Mass Index; EU-TIRADS, Thyroid Imaging Reporting Data System by European Thyroid Association; KTA-TIRADS, Thyroid Imaging Reporting Data System by Korean Thyroid Association; ROC, Receiver Operating Characteristic; RSS, Risk Stratification System; TSH, Thyroid Stimulating Hormone; TN, Thyroid Nodule; TC, Thyroid Cancer; US, Ultrasound.

## References

[1] Guth S Theune U Aberle J Galach A Bamberger CM. Very high prevalence of thyroid nodules detected by high frequency (13 MHz) ultrasound examination. Eur J Clin Invest (2009) 39(8):699–706. doi: 10.1111/j.1365-2362.2009.02162.x 19601965

[2] Ferlay J Colombet M Soerjomataram I Mathers C Parkin DM Pineros M. Estimating the global cancer incidence and mortality in 2018: GLOBOCAN sources and methods. Int J Cancer (2019) 144(8):1941–53. doi: 10.1002/ijc.31937 30350310

[3] Moon JH Hyun MK Lee JY Shim JI Kim TH Choi HS. Prevalence of thyroid nodules and their associated clinical parameters: a large-scale, multicenter-based health checkup study. Korean J Intern Med (2018) 33(4):753–62. doi: 10.3904/kjim.2015.273 PMC6030422 28859466

[4] Singh Ospina N Iniguez-Ariza NM Castro MR. Thyroid nodules: diagnostic evaluation based on thyroid cancer risk assessment. BMJ (2020) 368:l6670. doi: 10.1136/bmj.l6670 31911452

[5] Grani G Sponziello M Pecce V Ramundo V Durante C. Contemporary thyroid nodule evaluation and management. J Clin Endocrinol Metab (2020) 105(9):2869–83. doi: 10.1210/clinem/dgaa322 PMC7365695 32491169

[6] Ahn HS Kim HJ Welch HG. Korea’s thyroid-cancer “epidemic”–screening and overdiagnosis. N Engl J Med (2014) 371(19):1765–7. doi: 10.1056/NEJMp1409841 25372084

[7] Vaccarella S Franceschi S Bray F Wild CP Plummer M Dal Maso L. Worldwide thyroid-cancer epidemic? the increasing impact of overdiagnosis. N Engl J Med (2016) 375(7):614–7. doi: 10.1056/NEJMp1604412 27532827

[8] Tessler FN Middleton WD Grant EG Hoang JK Berland LL Teefey SA. ACR thyroid imaging, reporting and data system (TI-RADS): White paper of the ACR TI-RADS committee. J Am Coll Radiol (2017) 14(5):587–95. doi: 10.1016/j.jacr.2017.01.046 28372962

[9] Russ G Bonnema SJ Erdogan MF Durante C Ngu R Leenhardt L. European Thyroid association guidelines for ultrasound malignancy risk stratification of thyroid nodules in adults: The EU-TIRADS. Eur Thyroid J (2017) 6(5):225–37. doi: 10.1159/000478927 PMC5652895 29167761

[10] Ha EJ Na DG Moon WJ Lee YH Choi N. Diagnostic performance of ultrasound-based risk-stratification systems for thyroid nodules: Comparison of the 2015 American thyroid association guidelines with the 2016 Korean thyroid Association/Korean society of thyroid radiology and 2017 American college of radiology guidelines. Thyroid (2018) 28(11):1532–7. doi: 10.1089/thy.2018.0094 30311862

[11] Zhang WB Xu HX Zhang YF Guo LH Xu SH Zhao CK. Comparisons of ACR TI-RADS, ATA guidelines, Kwak TI-RADS, and KTA/KSThR guidelines in malignancy risk stratification of thyroid nodules. Clin Hemorheol Microcirc (2020) 75(2):219–32. doi: 10.3233/CH-190778 31929154

[12] Middleton WD Teefey SA Reading CC Langer JE Beland MD Szabunio MM. Comparison of performance characteristics of American college of radiology TI-RADS, Korean society of thyroid radiology TIRADS, and American thyroid association guidelines. AJR Am J Roentgenol (2018) 210(5):1148–54. doi: 10.2214/AJR.17.18822 29629797

[13] Kim PH Yoon HM Hwang J Lee JS Jung AY Cho YA. Diagnostic performance of adult-based ATA and ACR-TIRADS ultrasound risk stratification systems in pediatric thyroid nodules: a systematic review and meta-analysis. Eur Radiol (2021) 31(10):7450–63. doi: 10.1007/s00330-021-07908-8 33864505

[14] Kuru B Kefeli M Danaci M. Comparison of 5 thyroid ultrasound stratification systems for differentiation of benign and malignant nodules and to avoid biopsy using histology as reference standard. Endocr Pract (2021) 27(11):1093–9. doi: 10.1016/j.eprac.2021.04.411 33930581

[15] Liu J Guo Y Xiao J Chen L Liang Z. Comparison of the efficacy and safety of the American thyroid association guidelines and American college of radiology TI-RADS. Endocr Pract (2021) 27(7):661–7. doi: 10.1016/j.eprac.2020.11.013 34250908

[16] Suteau V Munier M Briet C Rodien P. Sex bias in differentiated thyroid cancer. Int J Mol Sci (2021) 22(23):12992. doi: 10.3390/ijms222312992 34884794 PMC8657786

[17] Panagiotou G Komninou D Anagnostis P Linardos G Karoglou E Somali M. Association between lifestyle and anthropometric parameters and thyroid nodule features. Endocrine (2017) 56(3):560–7. doi: 10.1007/s12020-017-1285-6 28390011

[18] Miranda-Filho A Lortet-Tieulent J Bray F Cao B Franceschi S Vaccarella S. Thyroid cancer incidence trends by histology in 25 countries: a population-based study. Lancet Diabetes Endocrinol (2021) 9(4):225–34. doi: 10.1016/S2213-8587(21)00027-9 33662333

[19] Haugen BR Alexander EK Bible KC Doherty GM Mandel SJ Nikiforov YE. 2015 American Thyroid association management guidelines for adult patients with thyroid nodules and differentiated thyroid cancer: The American thyroid association guidelines task force on thyroid nodules and differentiated thyroid cancer. Thyroid (2016) 26(1):1–133. doi: 10.1089/thy.2015.0020 26462967 PMC4739132

[20] Shin JH Baek JH Chung J Ha EJ Kim JH Lee YH. Ultrasonography diagnosis and imaging-based management of thyroid nodules: Revised Korean society of thyroid radiology consensus statement and recommendations. Korean J Radiol (2016) 17(3):370–95. doi: 10.3348/kjr.2016.17.3.370 PMC4842857 27134526

[21] Xia Y Wang L Jiang Y Dai Q Li X Li W. Sonographic appearance of primary thyroid lymphoma-preliminary experience. PloS One (2014) 9(12):e114080. doi: 10.1371/journal.pone.0114080 25474402 PMC4256385

[22] Sipos JA. Advances in ultrasound for the diagnosis and management of thyroid cancer. Thyroid (2009) 19(12):1363–72. doi: 10.1089/thy.2009.1608 20001718

[23] Leenhardt L Erdogan MF Hegedus L Mandel SJ Paschke R Rago T. 2013 European Thyroid association guidelines for cervical ultrasound scan and ultrasound-guided techniques in the postoperative management of patients with thyroid cancer. Eur Thyroid J (2013) 2(3):147–59. doi: 10.1159/000354537 PMC4017749 24847448

[24] Asteria C Giovanardi A Pizzocaro A Cozzaglio L Morabito A Somalvico F. US-Elastography in the differential diagnosis of benign and malignant thyroid nodules. Thyroid (2008) 18(5):523–31. doi: 10.1089/thy.2007.0323 18466077

[25] Davies L Welch HG. Current thyroid cancer trends in the united states. JAMA Otolaryngol Head Neck Surg (2014) 140(4):317–22. doi: 10.1001/jamaoto.2014.1 24557566

[26] Moleti M Sturniolo G Di Mauro M Russo M Vermiglio F. Female reproductive factors and differentiated thyroid cancer. Front Endocrinol (Lausanne) (2017) 8:111. doi: 10.3389/fendo.2017.00111 28588554 PMC5440523

[27] Bertakis KD Azari R. Patient gender differences in the prediction of medical expenditures. J Womens Health (Larchmt) (2010) 19(10):1925–32. doi: 10.1089/jwh.2009.1448 20831429

[28] Wang Y Hunt K Nazareth I Freemantle N Petersen I. Do men consult less than women? an analysis of routinely collected UK general practice data. BMJ Open (2013) 3(8):e003320. doi: 10.1136/bmjopen-2013-003320 PMC3753483 23959757

[29] Campanella P Ianni F Rota CA Corsello SM Pontecorvi A. Quantification of cancer risk of each clinical and ultrasonographic suspicious feature of thyroid nodules: a systematic review and meta-analysis. Eur J Endocrinol (2014) 170(5):R203–211. doi: 10.1530/EJE-13-0995 24536085

[30] Ohtsuru A Takahashi H Kamiya K. Incidence of thyroid cancer among children and young adults in fukushima, Japan-reply. JAMA Otolaryngol Head Neck Surg (2019) 145(8):770. doi: 10.1001/jamaoto.2019.1102 31194244

[31] Vergamini LB Frazier AL Abrantes FL Ribeiro KB Rodriguez-Galindo C. Increase in the incidence of differentiated thyroid carcinoma in children, adolescents, and young adults: a population-based study. J Pediatr (2014) 164(6):1481–5. doi: 10.1016/j.jpeds.2014.01.059 24630354

[32] Lamartina L Leboulleux S Schlumberger M. Thyroid cancer incidence in children and adolescents. Lancet Diabetes Endocrinol (2021) 9(3):128–9. doi: 10.1016/S2213-8587(20)30430-7 33482108

[33] Bray F Ferlay J Soerjomataram I Siegel RL Torre LA Jemal A. Global cancer statistics 2018: GLOBOCAN estimates of incidence and mortality worldwide for 36 cancers in 185 countries. CA Cancer J Clin (2018) 68(6):394–424. doi: 10.3322/caac.21492 30207593

[34] Choi JS Nam CM Kim EK Moon HJ Han KH Kwak JY. Evaluation of serum thyroid-stimulating hormone as indicator for fine-needle aspiration in patients with thyroid nodules. Head Neck (2015) 37(4):498–504. doi: 10.1002/hed.23616 24435826

[35] Na DG Baek JH Sung JY Kim JH Kim JK Choi YJ. Thyroid imaging reporting and data system risk stratification of thyroid nodules: Categorization based on solidity and echogenicity. Thyroid (2016) 26(4):562–72. doi: 10.1089/thy.2015.0460 26756476

[36] Ha EJ Moon WJ Na DG Lee YH Choi N Kim SJ. A multicenter prospective validation study for the Korean thyroid imaging reporting and data system in patients with thyroid nodules. Korean J Radiol (2016) 17(5):811–21. doi: 10.3348/kjr.2016.17.5.811 PMC5007410 27587972

[37] Moon WJ Jung SL Lee JH Na DG Baek JH Lee YH. Benign and malignant thyroid nodules: US differentiation–multicenter retrospective study. Radiology (2008) 247(3):762–70. doi: 10.1148/radiol.2473070944 18403624

[38] Grant EG Tessler FN Hoang JK Langer JE Beland MD Berland LL. Thyroid ultrasound reporting lexicon: White paper of the ACR thyroid imaging, reporting and data system (TIRADS) committee. J Am Coll Radiol (2015) 12(12 Pt A):1272–9. doi: 10.1016/j.jacr.2015.07.011 26419308

[39] Kim SY Na DG Paik W. Which ultrasound image plane is appropriate for evaluating the taller-than-wide sign in the risk stratification of thyroid nodules? Eur Radiol (2021) 31(10):7605–13. doi: 10.1007/s00330-021-07936-4 33855586

[40] Zhou J Yin L Wei X Zhang S Song Y Luo B. 2020 Chinese Guidelines for ultrasound malignancy risk stratification of thyroid nodules: the c-TIRADS. Endocrine (2020) 70(2):256–79. doi: 10.1007/s12020-020-02441-y 32827126

[41] Kwak JY Jung I Baek JH Baek SM Choi N Choi YJ. Image reporting and characterization system for ultrasound features of thyroid nodules: multicentric Korean retrospective study. Korean J Radiol (2013) 14(1):110–7. doi: 10.3348/kjr.2013.14.1.110 PMC3542293 23323040

[42] Papini E Guglielmi R Bianchini A Crescenzi A Taccogna S Nardi F. Risk of malignancy in nonpalpable thyroid nodules: predictive value of ultrasound and color-Doppler features. J Clin Endocrinol Metab (2002) 87(5):1941–6. doi: 10.1210/jcem.87.5.8504 11994321

[43] Frates MC Benson CB Doubilet PM Cibas ES Marqusee E. Can color Doppler sonography aid in the prediction of malignancy of thyroid nodules? J Ultrasound Med (2003) 22(2):127–31. doi: 10.7863/jum.2003.22.2.127 12562117

[44] Rago T Vitti P Chiovato L Mazzeo S De Liperi A Miccoli P. Role of conventional ultrasonography and color flow-doppler sonography in predicting malignancy in ‘cold’ thyroid nodules. Eur J Endocrinol (1998) 138(1):41–6. doi: 10.1530/eje.0.1380041 9461314

[45] Appetecchia M Solivetti FM. The association of colour flow Doppler sonography and conventional ultrasonography improves the diagnosis of thyroid carcinoma. Horm Res (2006) 66(5):249–56. doi: 10.1159/000096013 17016052

[46] Ma JJ Ding H Xu BH Xu C Song LJ Huang BJ. Diagnostic performances of various gray-scale, color Doppler, and contrast-enhanced ultrasonography findings in predicting malignant thyroid nodules. Thyroid (2014) 24(2):355–63. doi: 10.1089/thy.2013.0150 23978252

[47] Moon HJ Kwak JY Kim MJ Son EJ Kim EK. Can vascularity at power Doppler US help predict thyroid malignancy? Radiology (2010) 255(1):260–9. doi: 10.1148/radiol.09091284 20308462

[48] Sigrist RMS Liau J Kaffas AE Chammas MC Willmann JK. Ultrasound elastography: Review of techniques and clinical applications. Theranostics (2017) 7(5):1303–29. doi: 10.7150/thno.18650 PMC5399595 28435467

[49] Bojunga J Herrmann E Meyer G Weber S Zeuzem S Friedrich-Rust M. Real-time elastography for the differentiation of benign and malignant thyroid nodules: a meta-analysis. Thyroid (2010) 20(10):1145–50. doi: 10.1089/thy.2010.0079 20860422

[50] Tessler FN. Thyroid nodules and real estate: Location matters. Thyroid (2020) 30(3):349–50. doi: 10.1089/thy.2020.0090 32031059

[51] Persichetti A Di Stasio E Coccaro C Graziano F Bianchini A Di Donna V. Inter- and intraobserver agreement in the assessment of thyroid nodule ultrasound features and classification systems: A blinded multicenter study. Thyroid (2020) 30(2):237–42. doi: 10.1089/thy.2019.0360 31952456

