# Supplementary material for: Development and validation of a novel diagnostic tool for predicting the malignancy probability of thyroid nodules: A retrospective study based on clinical, B-mode, color doppler and elastographic ultrasonographic characteristics
Source: Front Endocrinol (Lausanne). 2022 Sep 20;13:966572. doi: 10.3389/fendo.2022.966572 (PMC9530571; doi:10.3389/fendo.2022.966572)
Supplement: Supplementary file 1 [file Table_1.docx]

**Supplement Table** Numbers of benign and malignant nodules

| Pathology | | Number |
| --- | --- | --- |
| Benign | Nodular goiter | 313 |
|  | Hashimoto thyroiditis | 39 |
|  | Follicular adenoma | 22 |
| Malignant | Papillary thyroid carcinoma | 617 |
|  | Medullary thyroid carcinoma | 15 |
|  | Follicular thyroid carcinoma | 10 |
